# Specific Distribution of Phosphatidylglycerol to Photosystem Complexes in the Thylakoid Membrane

**DOI:** 10.3389/fpls.2017.01991

**Published:** 2017-11-20

**Authors:** Koichi Kobayashi, Kaichiro Endo, Hajime Wada

**Affiliations:** Department of Life Sciences, Graduate School of Arts and Sciences, The University of Tokyo, Tokyo, Japan

**Keywords:** thylakoid membrane, monogalactosyldiacylglycerol, digalactosyldiacylglycerol, sulfoquinovosyldiacylglycerol, phosphatidylglycerol, lipid bilayer, photosystem I, photosystem II

## Abstract

The thylakoid membrane is the site of photochemical and electron transport reactions of oxygenic photosynthesis. The lipid composition of the thylakoid membrane, with two galactolipids, one sulfolipid, and one phospholipid, is highly conserved among oxygenic photosynthetic organisms. Besides providing a lipid bilayer matrix, thylakoid lipids are integrated in photosynthetic complexes particularly in photosystems I and II and play important roles in electron transport processes. Thylakoid lipids are differentially allocated to photosynthetic complexes and the lipid bilayer fraction, but distribution of each lipid in the thylakoid membrane is unclear. In this study, based on published crystallographic and biochemical data, we estimated the proportions of photosynthetic complex-associated and bilayer-associated lipids in thylakoid membranes of cyanobacteria and plants. The data suggest that ∼30 mol% of phosphatidylglycerol (PG), the only major phospholipid in thylakoid membranes, is allocated to photosystem complexes, whereas glycolipids are mostly distributed to the lipid bilayer fraction and constitute the membrane lipid matrix. Because PG is essential for the structure and function of both photosystems, PG buried in these complexes might have been selectively conserved among oxygenic phototrophs. The specific and substantial allocation of PG to the deep sites of photosystems may need a unique mechanism to incorporate PG into the complexes possibly in coordination with the synthesis of photosynthetic proteins and pigments.

## Introduction

The thylakoid membrane is the site of photochemical and electron transport reactions in oxygenic phototrophs. In thylakoid membranes, glycerolipids form a bilayer matrix, in which photosynthetic protein–cofactor complexes such as photosystem I (PSI), photosystem II (PSII), cytochrome (Cyt) *b*_6_/*f* complex and ATP synthase are embedded. The hydrophobic lipid bilayer prevents free diffusion of ions and allows for creating a proton gradient while enabling lateral diffusion of the photosynthetic complexes and the mobile electron carriers for efficient electron transport reactions.

The lipid composition of the thylakoid membrane is highly conserved among oxygenic phototrophs (Murata et al., 1981; Mendiola-Morgenthaler et al., 1985; Dorne et al., 1990). Galactolipids, monogalactosyldiacylglycerol (MGDG) and digalactosyldiacylglycerol (DGDG), account for ∼50 and ∼30 mol% of total thylakoid lipids, respectively. Thylakoids contain another unique glycolipid, sulfoquinovosyldiacylglycerol (SQDG), which constitutes ∼10% of the total lipids. The remaining ∼10% is phosphatidylglycerol (PG), the only major phospholipid in thylakoids.

Each lipid class has a different physicochemical property depending on the nature of its head group. MGDG and DGDG are neutral lipids with uncharged polar head groups. MGDG has a cone-like shape with a small galactose head group and flexible poly-unsaturated fatty acid tails and thus tends to form a non-bilayer lipid phase in mixture with water (Shipley et al., 1973; Jouhet, 2013). By contrast, DGDG has a more bulky head group with two galactoses, which gives DGDG a cylindrical shape and allows for forming a bilayer lamellar phase. The MGDG/DGDG ratio strongly affects the phase behavior of the lipid bilayer and may be important for the structure and stability of the thylakoid membrane (Murphy, 1982; Demé et al., 2014). SQDG and PG, which are both bilayer lipids, have negative charge at neutral pH and are classified as acidic lipids. PG and SQDG are in part functionally redundant in both cyanobacteria and plant chloroplasts, as we describe later, presumably because of their common acidic properties in polar head groups.

Besides providing a lipid bilayer matrix, thylakoid lipids are integrated in PSII (Loll et al., 2005, 2007; Sakurai et al., 2006; Umena et al., 2011; Wei et al., 2016), Cyt *b*_6_/*f* (Kurisu et al., 2003; Stroebel et al., 2003; Baniulis et al., 2009; Hasan et al., 2011), PSI (Jordan et al., 2001; Kubota et al., 2010; Qin et al., 2015; Mazor et al., 2017), and light-harvesting complexes (LHCs) (Liu et al., 2004; Standfuss et al., 2005; Wei et al., 2016) and play important roles in electron transport processes (Kobayashi et al., 2016b). Because these photosynthetic complexes account for large quantities of the thylakoid membrane, substantial amounts of lipids would be allocated to the complexes as structural components. Based on published crystallographic and biochemical data, we estimated the amount of photosynthetic complex-associated lipids and lipid composition in the thylakoid lipid bilayer. The results indicated a preferential distribution of PG to photosystems and a minor contribution to the thylakoid lipid bilayer in plant chloroplasts and cyanobacteria.

## Distribution of Lipids in the Thylakoid Membrane in Cyanobacteria

Jordan et al. (2001) first revealed the crystal structure of PSI from *Thermosynechococcus elongatus* at 2.5-Å resolution. In the PSI trimer, 1 MGDG and 3 PG molecules per monomer were identified. The same lipid configuration was observed in the PSI crystal structure of *Synechocystis* sp. PCC 6803 (Mazor et al., 2014). Meanwhile, crystallographic analysis of the PSII dimer from *Thermosynechococcus vulcanus*, the species closely related to *T. elongatus*, at 1.9-Å resolution identified 20 lipid molecules (6 MGDGs, 5 DGDGs, 4 SQDGs, and 5 PGs) and 3 unknown diglycerides per monomer (Umena et al., 2011). A similar lipid composition was revealed by lipid analysis of PSII dimers purified from *T. vulcanus* and *Synechocystis* sp. PCC 6803 (Sakurai et al., 2006), which supports the validity of these results.

**FIGURE 1:**
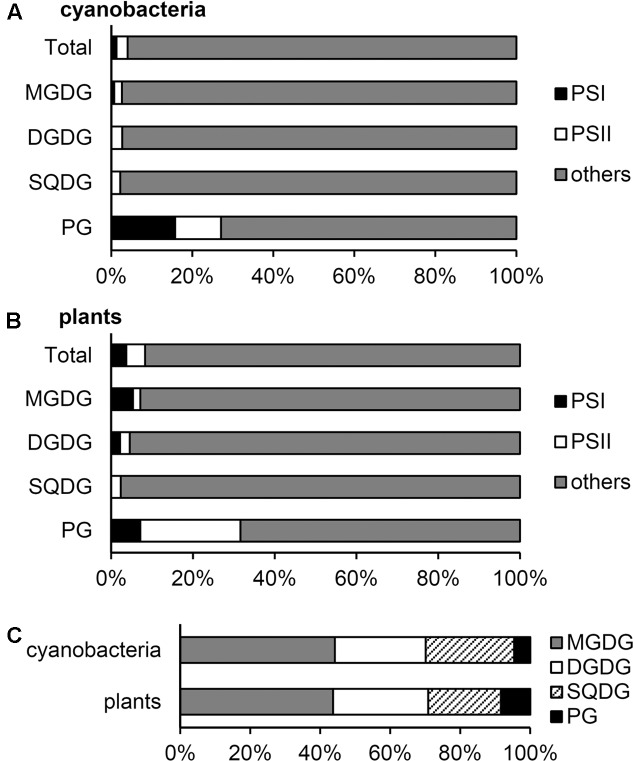
Distribution of glycerolipids to photosystems and other fractions in the thylakoid membrane. **(A,B)** Distribution (mol%) of each lipid to photosystem I (PSI), photosystem II (PSII) and other thylakoid fractions in cyanobacteria **(A)** and plants **(B)**. **(C)** Lipid composition (mol%) of the thylakoid fraction outside photosystems. Lipid compositions are computed from the data in **Table 1**.

From the data in Sakurai et al. (2006), we computed the molar ratio of each lipid to chlorophyll (Chl) in the thylakoid fraction from *T. vulcanus* (**Table 1**). Because of no available data for the total PSI and PSII content in *Thermosynechococcus* species, in this study, we used the data for PSI (7.5 mmol/mol Chl) and PSII (3.2 mmol/mol Chl) content in another thermophilic cyanobacterium *Synechococcus lividus* (Melis and Brown, 1980) for a rough estimation. Similar content in *Synechocystis* sp. PCC 6714 (Fujita and Murakami, 1987; Murakami et al., 1997) supports the validity of this assumption. In combination with the crystallographic data (Jordan et al., 2001; Umena et al., 2011), these biochemical data allowed us to estimate 1.3 and 2.8 mol% of total thylakoid lipids allocated to PSI and PSII, respectively (**Figure 1A**). Of note, ∼30% of total PG molecules is associated with photosystems, whereas only a small portion (∼3%) is allocated to photosystems for other lipid classes. As a result, PG accounts for only 4.6 mol% of total lipids outside photosystems, presumably mainly in the lipid bilayer fraction. These data demonstrate that PG, the only phospholipid in cyanobacteria, is preferentially allocated to photosystem complexes, whereas glycolipids predominantly constitute the lipid bilayer of the thylakoid membrane. We should note that because the PSI-to-PSII ratios in cyanobacteria flexibly change from ∼0.5 to ∼5 depending on growth conditions, particularly light quality and quantity (Fujita and Murakami, 1987; Samson et al., 1994; Murakami et al., 1997), the proportion of PG in photosystems may also range from ∼45 to ∼23 mol% of total thylakoid PG.

**Table 1:** Lipid content in the thylakoid membrane and photosystem complexes.

				Thylakoid	PSI	PSII	Other
Cyanobacteria		PSI		7.52	–	–	–
		PSII		3.24	–	–	–
	Lipids		Total	2350	30.1 (4)	64.8 (20)	2230
			MGDG	1020	7.52 (1)	19.4 (6)	995
			DGDG	602	0 (0)	16.2 (5)	586
			SQDG	583	0 (0)	13.0 (4)	570
			PG	143	22.6 (3)	16.2 (5)	104
Plants		PSI		2.25	–	–	–
		PSII		2.99	–	–	–
	Lipids		Total	1950	72.0 (32)	89.7 (30)	1790
			MGDG	840	45.0 (20)	15.0 (5)	780
			DGDG	510	11.3 (5)	12.0 (4)	487
			SQDG	380	0 (0)	9.00 (3)	371
			PG	220	15.8 (7)	53.8 (18)	150


*Data are expressed as mmol mol^-*1*^ chlorophyll. Data are derived or calculated from Melis and Brown (1980) for the content of photosystem I (PSI) and photosystem II (PSII) in a cyanobacterium (*Synechococcus lividus*), Sakurai et al. (2006) for each lipid content in the thylakoid membrane of a cyanobacterium (*Thermosynechococcus vulcanus*), Kirchhoff et al. (2002) for the content of PSI, PSII and each lipid in the thylakoid membrane of a plant (spinach). The figures in parentheses denote numbers of each lipid in PSI and PSII deduced from crystallographic analyses of PSI from *Thermosynechococcus elongatus* (Jordan et al., 2001), PSII from *T. vulcanus* (Umena et al., 2011), PSI-light harvesting complex (LHC) I from pea (Mazor et al., 2017), and PSII–LHCII from spinach (Wei et al., 2016).*

## Distribution of Lipids in the Thylakoid Membrane in Plants

Plants contain LHC antenna complexes in addition to core complexes in both PSI and PSII complexes. The crystal structure of the PSI–LHCI complex from pea at 2.8-Å resolution includes 10 lipid molecules (3 MGDGs, 1 DGDG, and 6 PGs) per monomer (Qin et al., 2015). Similar to *T. elongatus* PSI (Jordan et al., 2001), 1 MGDG, 1 DGDG, and 3 PG molecules were associated with the PSI core in pea. The more recent analysis of the pea PSI–LHCI complex at 2.6-Å resolution identified 27 lipid molecules (19 MGDGs, 4 DGDGs, and 4 PGs), mostly located between PSI and LHCI, in addition to the five lipids in the core region (Mazor et al., 2017).

Single-particle cryo-electron microscopic analysis of the C_2_S_2_-type PSII–LHCII supercomplex (C; PSII core complex, S; strongly associated LHCII trimer) from spinach at 3.2-Å resolution revealed 21 lipid molecules per monomeric complex (Wei et al., 2016). The lipid content (5 MGDGs, 4 DGDGs, 3 SQDGs, and 4 PGs) in the spinach PSII core is similar to that in *T. vulcanus* (Umena et al., 2011). In addition, 5 PG molecules were associated with the LHCII complex composed of 1 trimeric LHCII with 3 PGs, 1 monomeric CP26 with 1 PG, and 1 monomeric CP29 with 1 PG. X-ray crystallography analyses of the LHCII trimer from spinach at 2.7 Å (Liu et al., 2004) and from pea at 2.5 Å (Standfuss et al., 2005) both identified one PG molecule per monomer, which is consistent with the data in the C_2_S_2_-type PSII–LHCII supercomplex (Wei et al., 2016). By contrast, 2 DGDG molecules per monomer observed at the periphery of the crystallized LHCII trimers (Liu et al., 2004; Standfuss et al., 2005) were missing in the C_2_S_2_-type supercomplex. Because binding sites of DGDG differed between spinach and pea data, these DGDGs may be integrated into the LHCII trimer during crystallization processes. Besides the C_2_S_2_-type complex, other forms of the PSII–LHCII supercomplex such as C_2_S_2_M_2_ type (M; moderately associated LHCII trimer) have been identified in plant thylakoids (Pan et al., 2013). Biochemical analysis revealed that a single PSII–LHCII unit contains 250 Chls on average (Ghirardi et al., 1986), which would correspond to 4 major LHCII trimers (12 subunits in total) and 3 minor monomeric LHCIIs, presumably CP24, CP26, and CP29, per PSII monomer (Peter and Thornber, 1991; Pan et al., 2013). This estimation is consistent with the 3.8-times higher amount of the LHCII trimer than the PSII monomer on a molar basis in spinach thylakoids (Kirchhoff et al., 2002). From these data, we assumed that PSII–LHCII complexes contain a total of 30 lipids (5 MGDGs, 4 DGDGs, 3 SQDGs, and 18 PGs) per PSII monomer, on average.

Although the relative abundance of PSI, PSII, and LHCII in plants greatly differs according to growth conditions, in this study, we estimated lipid content in photosystem complexes based on the data in spinach thylakoids (**Table 1**) (Kirchhoff et al., 2002). The estimation revealed that PSI–LHCI and PSII–LHCII contain 3.7 and 4.6 mol%, respectively, total lipids in the thylakoid membrane (**Figure 1B**). As in cyanobacteria, ∼30 mol% of total PG in the thylakoid membrane is allocated to photosystems in plant chloroplasts, and thus the contribution of PG to other lipid fractions (8.4 mol%) is lower than that of SQDG (20.7 mol%) (**Figure 1C**).

## Distribution of Lipids in Other Thylakoid Fractions

The Cyt *b*_6_/*f* complex mediates electron transfer from PSII to PSI and generates an electrochemical proton gradient across the thylakoid membrane together with water splitting in PSII. The crystal structure of the dimeric Cyt *b*_6_/*f* complex has been determined for *Chlamydomonas reinhardtii* (Stroebel et al., 2003), *Mastigocladus laminosus* (Kurisu et al., 2003) and *Nostoc* sp. PCC 7120 (Baniulis et al., 2009). From these data, one SQDG molecule was identified with two synthetic phosphatidylcholine and four detergents per monomer. In addition, mass spectroscopy analysis of spinach Cyt *b*_6_/*f* detected MGDG, DGDG, and PG along with SQDG (Hasan et al., 2011). Although two neutral lipids at the lumenal side were modeled as MGDGs in the *C. reinhardtii* Cyt *b*_6_/*f* (Stroebel et al., 2003), one may be a DGDG molecule based on the mass spectroscopy data. Furthermore, the presence of stoichiometric amounts of PG and SQDG in spinach Cyt *b*_6_/*f* suggests an equal amount of these anionic lipids in the structure (Hasan et al., 2011). Considering the content of Cyt *b*_6_/*f* of ∼1.0 mmol/mol Chl in plants (Kirchhoff et al., 2002; Schöttler and Toth, 2014) and ∼5.0 mmol/mol Chl in cyanobacteria (Fujita and Murakami, 1987), we roughly estimated that 0.3 ∼ 0.9 mol% of total thylakoid lipids are integrated in the Cyt *b*_6_/*f* complexes with stoichiometry of 1 MGDG, 1 DGDG, 1 SQDG, and 1 PG per monomer.

For ATP synthesis, ATP synthase uses a proton gradient across the thylakoid membrane. In animals, cardiolipin (diphosphatidylglycerol), the anionic lipid produced from PG and CDP-diacylglycerol, is an essential component of mitochondrial ATP synthase (Santiago et al., 1973; Laird et al., 1986). Computer simulations demonstrated that cardiolipin specifically and transiently interacts with the rotating c-ring of yeast ATP synthase (Duncan et al., 2016). In the thylakoid membrane, containing no cardiolipin, the anionic lipid PG or SQDG may be specifically associated with ATP synthase, although the lipid content in the thylakoid ATP synthase remains undetermined.

Considering the predominant amount of photosynthetic electron transport complexes in the thylakoid membrane (Yu et al., 1994; Herranen et al., 2004), lipids that do not integrate in these major complexes would be mostly allocated to the thylakoid lipid bilayer. We estimated that 92–95 mol% of the thylakoid lipid bilayer is composed of glycolipids (**Figure 1C**).

## Role of PG in Photosystems

In general, the primary role of membrane glycerolipids is to construct the lipid bilayer. In fact, most glycolipids in thylakoid are distributed to the lipid bilayer fraction as building blocks (**Figure 1**). However, our estimation revealed that ∼30 mol% of PG in the thylakoid membrane is allocated to photosystem complexes as a structural component in both cyanobacteria and plant chloroplasts, and the quantitative contribution of PG to the thylakoid lipid bilayer is low (5–8 mol%). Because loss of PG severely impairs photosynthetic electron transport in all oxygenic phototrophs examined to date (Kobayashi et al., 2016b), this lipid would specifically serve as an essential component of photosystems.

In the crystal structure of *T. vulcanus* PSII, all 5 PG molecules are deeply buried near the reaction center (Umena et al., 2011). Short-term depletion (3–7 days) of PG in *Synechocystis* sp. PCC 6803 cells caused loss of ∼3 PG molecules from PSII, accompanied by impaired electron transport from the primary (*Q*_A_) to the secondary plastoquinone (*Q*_B_) and destabilized oxygen-evolving complex (Sakurai et al., 2003, 2007). Moreover, site-directed mutations of PG-associated amino-acid residues of the D1 protein, which caused loss of about 1 PG molecule from the PSII complex, decreased electron transport activity and disordered PSII structures (Endo et al., 2015). These data demonstrate the essential roles of PSII-associated PG in structures and functions of the complex.

In contrast to the rapid inhibition of PSII by PG deficiency, PSI is affected only after a longer period (>2 weeks) of PG depletion (Domonkos et al., 2004), which may reflect more stable association of PG with PSI. Three PG molecules are integrated in the crystal structure of *T. elongatus* PSI (Jordan et al., 2001). One is buried near the reaction center, and the other two locate at the periphery. Decreased PSI activity and increased monomeric PSI complexes after long-term PG deficiency suggest an important role of PG in the activity and structural organization of PSI (Domonkos et al., 2004).

In plants, PG molecules are associated with the LHCI and LHCII complexes in addition to the photosystem core complexes (Liu et al., 2004; Standfuss et al., 2005; Qin et al., 2015; Wei et al., 2016; Mazor et al., 2017). Degradation of PG by phospholipase A2 treatment disassembled the LHCII trimer into the monomeric form (Nussberger et al., 1993; Kim et al., 2007). Moreover, PG deficiency in a *C. reinhardtii* mutant decreased trimeric LHCII level, but PG supplementation to the mutant partially recovered the phenotype (Dubertret et al., 1994). These findings indicate a crucial role of PG in structural organization of the LHCII trimer. In addition, in the pea PSI–LHCI complex, several PG molecules are located between PSI and LHCI with MGDGs and DGDGs (Mazor et al., 2017). Because lack of any of these lipids caused disorganization of PSI–LHCI complexes in *Arabidopsis thaliana* mutants (Ivanov et al., 2006; Kobayashi et al., 2013, 2016a), the lipid cluster observed between PSI and LHCI may specifically function to maintain the complex structure.

The preferential allocation of PG to the deep sites of both photosystems despite the low abundance of PG in the thylakoid lipid bilayer suggests a requirement of specific mechanisms to deliver PG molecules within the protein complexes. Kopečná et al. (2015) reported that loss of PG in the *Synechocystis* sp. PCC 6803 mutant strongly impaired synthesis of PSI proteins and Chls. The authors proposed that a PG-containing membrane microdomain may be required for the synthesis of these components (Kopečná et al., 2015). Considering the low amount of PG in the thylakoid lipid bilayer, PG distribution may be restricted to a specific microdomain in the thylakoid membrane, where assembly of PG to protein–pigment complexes may take place along with synthesis of Chls and PSI.

## Role of PG Outside Photosystems

Mutant analyses in *A. thaliana*, *C. reinhardtii* and several cyanobacteria revealed that loss of SQDG increased PG levels to maintain total anionic lipid content (Sato et al., 1995; Güler et al., 1996; Yu et al., 2002; Aoki et al., 2004; Endo et al., 2016). In *T. elongatus*, PG content was increased from 4 to 25 mol% by disruption of SQDG biosynthesis (Endo et al., 2016). Because photosystem-associated SQDG is only 3.7 mol% of the total SQDG level in *Thermosynechococcus* thylakoids (**Figure 1A**), PG produced in response to loss of SQDG would be mainly allocated to the lipid bilayer fraction. Although complete lack of SQDG alone has no or only weak effects in most photosynthetic organisms (Sato et al., 1995; Güler et al., 1996; Yu et al., 2002; Endo et al., 2016), a partial decrease in PG content along with lack of SQDG strongly impaired photosynthetic activity and growth (Güler et al., 1996; Yu and Benning, 2003; Endo et al., 2016). The result suggests an importance of maintained levels of total anionic lipids in thylakoids. Because SQDG is the major anionic lipid in the thylakoid lipid bilayer (**Figure 1C**), the main role of this lipid may be maintaining a certain level of anionic lipids in the membrane. By contrast, PG has more specific roles in photosystems. However, when SQDG content is decreased, PG content increases and would complement the role of SQDG in the lipid bilayer.

Under phosphate-limited conditions, PG content in *A. thaliana* decreased from ∼10 to ∼5 mol% of total membrane lipids, with SQDG content inversely increased from ∼6 to ∼11 mol% (Essigmann et al., 1998). Because photosystem-associated PG is indispensable for photosynthesis, PG molecules that are not integrated in the complexes may be preferentially degraded under phosphate limitation. This assumption is supported by the fact that phosphate limitation severely affected photosynthetic electron transport in PG-deficient *A. thaliana* mutants but not the wild type (Kobayashi et al., 2015). Thus, increased SQDG under phosphate limitation would mainly replace PG outside photosystem complexes. A similar relationship between PG and SQDG under phosphate limitation is observed in *S. elongatus* PCC 7942 (Güler et al., 1996) but not *Synechocystis* sp. PCC 6803 (Awai et al., 2007) or *T. elongatus* (Endo et al., 2016). PG content is substantially low in *T. elongatus* (∼4 mol%) as compared with *S. elongatus* (16.6 mol%) even under phosphate-sufficient conditions (Endo et al., 2016). Conversely, SQDG content is higher in *T. elongatus* (∼18 mol%) than *S. elongatus* (10.3 mol%). *T. elongatus* may already decrease PG content to a minimal level under phosphate-sufficient conditions and thus cannot further reduce PG content under limited phosphate. Use of glycolipids for the lipid bilayer reduces spending phosphate in the membrane, which would be advantageous for growth of photosynthetic organisms particularly under phosphate-limited conditions. The specific enrichment of PG in photosystems implies an indispensable role of this lipid, which presumably could not have been replaced by glycolipids during the long evolutionary processes of oxygenic photosynthetic organisms.

## Conclusion

In both plants and cyanobacteria, one third of total PG molecules in thylakoids is specifically integrated with photosystems to fulfill its essential role in photosynthesis. By contrast, galactolipids mainly constitute the thylakoid lipid bilayer with the anionic glycolipid SQDG, which reduces the use of phosphate in the thylakoid membrane. The specific enrichment of PG in photosystems implies a particular mechanism to assemble PG into the deep sites of the complexes, which should be evaluated in future studies.

## Author Contributions

KK conceived the study, analyzed data, and wrote the manuscript. KE analyzed data and complemented the writing. HW supervised the study and complemented the writing.

## Conflict of Interest Statement

The authors declare that the research was conducted in the absence of any commercial or financial relationships that could be construed as a potential conflict of interest.
